# STAT5B^N642H^ drives transformation of NKT cells: a novel mouse model for CD56^+^ T-LGL leukemia

**DOI:** 10.1038/s41375-019-0471-3

**Published:** 2019-04-09

**Authors:** Klara Klein, Agnieszka Witalisz-Siepracka, Barbara Maurer, Daniela Prinz, Gerwin Heller, Nicoletta Leidenfrost, Michaela Prchal-Murphy, Tobias Suske, Richard Moriggl, Veronika Sexl

**Affiliations:** 1 Institute of Pharmacology and Toxicology, University of Veterinary Medicine Vienna, Vienna, Austria; 2 Department of Medicine I, Medical University of Vienna, Vienna, Austria; 3 Institute of Animal Breeding and Genetics, University of Veterinary Medicine Vienna, Vienna, Austria; 4 Ludwig Boltzmann Institute for Cancer Research, Vienna, Austria; 5 Medical University of Vienna, Vienna, Austria

**Keywords:** Cancer models, Leukaemia

## To the Editor

The signal transducer and activator of transcription 5B (STAT5B), downstream of IL-15 signaling and Janus kinase (JAK)1, and 3-mediated activation, is a master regulator of development, survival, and function of innate and innate-like lymphocytes (including natural killer (NK) and NKT cells) [1–3]. Gain-of-function mutations in the SH2 domain of human STAT5B, especially STAT5B^N642H^, are associated with aggressive forms of CD56^+^ T cell (NKT) and NK cell lymphomas/leukemias [4–6]. We described a mouse model expressing human (h)STAT5B^N642H^ under the *Vav-1* promoter, which develops severe CD8^+^ T cell neoplasia [7]. Here, we explore the ability of hSTAT5B^N642H^ to serve as an oncogenic driver in innate lymphocyte neoplasms.

**Fig. 1:**
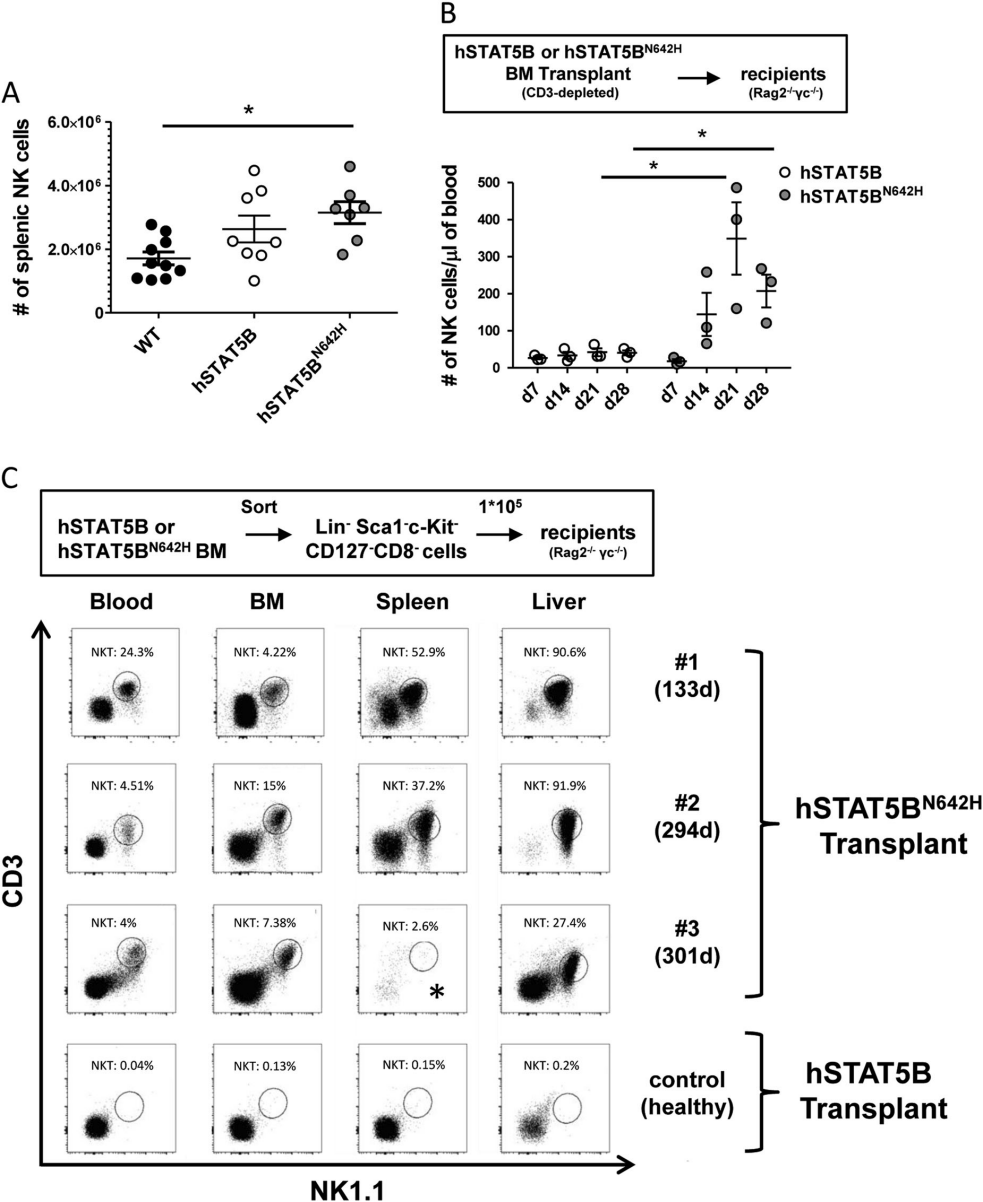
hSTAT5B^N642H^ drives limited NK cell expansion and gives rise to leukemia characterized by expansion of NKT cells. **a** Absolute numbers (#) of splenic NK cells (CD3^−^NK1.1^+^NKp46^+^) were determined in WT, hSTAT5B, and hSTAT5B^N642H^ mice by flow cytometry (*n* = 10 (WT), *n* = 8 (hSTAT5B), *n* = 7 (hSTAT5B^N642H^) pooled from four independent experiments). Symbols represent results from individual mice, horizontal lines indicate mean ± SEM. ^*^p < 0.05, one-way ANOVA. **b** CD3-depleted bone marrow (BM) from hSTAT5B or hSTAT5B^N642H^ mice was transplanted into Rag2^−/−^γc^−/−^ recipient mice (*n* = 3) and NK cell numbers were monitored in the blood weekly for 4 weeks by flow cytometry. One representative experiment from two independent experiments is shown. Symbols represent results from individual mice, horizontal lines indicate mean ± SEM. ^*^p < 0.05, unpaired *t*-test with Welch’s correction. **c** Lineage (Lin)^−^ Sca1^−^c-Kit^−^CD127^−^CD8^−^ cells were sorted from BM of hSTAT5B or hSTAT5B^N642H^ mice and 1 × 10^5^ cells were injected into Rag2^−/−^γc^−/−^ recipient mice (*n* = 3). Percentages of NKT cells (CD3^+^NK1.1^+^) among living cells in blood, BM, spleen, and liver were analyzed by flow cytometry from the diseased hSTAT5B^N642H^-transplanted recipient mice (#1–3) (days of survival after transplant are indicated in brackets) and a healthy hSTAT5B-transplanted control mouse. Dot plots are shown. We could not analyze NKT cell frequency in the spleen of mouse #3 (marked by *), due to limited quality of spleen tissue. Analysis was performed when the diseased mice reached the humane endpoint

We found an increase in absolute NK cell numbers in the spleen of T cell-diseased hSTAT5B^N642H^ transgenic compared to wild-type (WT) mice (Fig. 1a), despite the relative decrease of the proportion of NK cells among splenic lymphocytes (Fig. S1A), while nonmutant hSTAT5B control mice showed intermediate NK cell numbers (Fig. 1a, Fig. S1A). No significant differences in NK cell numbers were observed in the bone marrow (BM) between genotypes (Fig. S1B). In addition, both hSTAT5B and hSTAT5B^N642H^ mice showed a similar increase in the proportion of mature NK cells (CD27^−^CD11b^+^ and KLRG1^+^) in the spleen (Fig. S1C, D). These findings suggest that the enforced expression of nonmutant hSTAT5B is sufficient to boost NK cell maturation, which is not further enhanced by introducing the activating hSTAT5B mutation. We hypothesized that any phenotypic alterations affecting innate lymphocytes might be masked by the hSTAT5B^N642H^-driven aggressive CD8^+^ T cell disease established in hSTAT5B^N642H^ mice at the age of 6–8 weeks [7]. To explore the potential of hSTAT5B^N642H^ to promote NK cell expansion in vivo, we transplanted CD3-depleted BM from hSTAT5B^N642H^ or hSTAT5B mice in immune-deficient Rag2^−/−^γc^−/−^ recipient mice. Using this approach, we observed an enhanced expansion of NK cells in the blood of hSTAT5B^N642H^ compared to hSTAT5B-transplanted recipients over a time course of 4 weeks (Fig. 1b). Long-term analysis of NK cells in hSTAT5B^N642H^-transplanted mice was not possible due to expansion of residual CD8^+^ T cells (Fig. S1E, F), which forced us to terminate the experiment. However, after 4 weeks, increased numbers of NK cells were also detected in the spleen upon transplantation of CD3-depleted hSTAT5B^N642H^ compared to hSTAT5B BM (Fig. S1G).

To investigate long-term effects of hSTAT5B^N642H^ on innate lymphocytes, we sorted Lineage (Lin)^−^ (CD3/B220/Ter119/Gr1/CD11b) Sca1^−^c-Kit^−^CD127^−^CD8^−^ cells. This cellular fraction is devoid of hematopoietic stem cells, common lymphoid progenitors, and CD8^+^ T cells and was obtained from hSTAT5B^N642H^ or hSTAT5B BM for further transplantation into Rag2^−/−^γc^−/−^ recipient mice. We monitored NK and T cell numbers in the blood over a period of 5 weeks. Again, a modest increase but subsequent drop in NK cell numbers in mice transplanted with hSTAT5B^N642H^ compared to hSTAT5B BM cells was observed (Fig. S1H), whereas we failed to detect CD8^+^ T cells in the blood of the recipient animals (Fig. S1I). This indicated that our sorting strategy successfully eliminated CD8^+^ T cell disease, but did not allow us to uncover persistent hSTAT5B^N642H^-mediated NK cell expansion. Of note, after 4.5–10 months all three hSTAT5B^N642H^-recipient mice (#1–3) developed a rapidly progressing disease, characterized by expansion of CD3^+^NK1.1^+^ NKT cells in blood, BM, spleen, and liver (Fig. 1c, Table S1). Disease development was restricted to transplantation of mutant hSTAT5B cells and was not detected upon transplantation of hSTAT5B BM, despite following up these animals for 10 months.

**Fig. 2:**
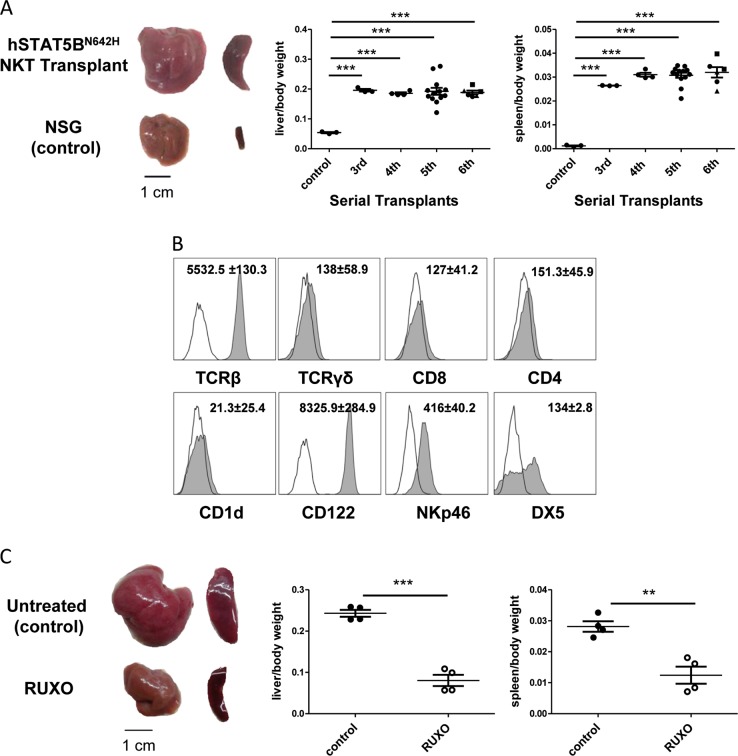
hSTAT5B^N642H^ induces leukemia of CD1d-independent NKp46^+^ NKT cells, which is serially transplantable and sensitive to Ruxolitinib treatment. **a** BM from NKT cell-diseased recipient #1, transplanted with sorted hSTAT5B^N642H^ BM cells, was serially transplanted into Rag2^−/−^γc^−/−^ or NSG-recipient mice for six rounds. Whole BM, containing 1 × 10^6^ transformed NKT cells, was transplanted. Of note, in the 6th serial transplant (ST) round a titration of the number of transplanted NKT cells was performed, with two mice each receiving BM containing 1 × 10^6^ (circle), 0.3 × 10^6^ (rectangle), or 0.1 ×10^6^ (triangle) NKT cells. Representative images of liver and spleen from an hSTAT5B^N642H^ NKT cell-transplanted (6th ST) compared to a non-transplanted NSG mouse are shown; line denotes 1 cm (left panel). Relative liver and spleen to body weights are shown for the 3rd to 6th ST (*n* = 3 (3rd ST), *n* = 4 (4th ST), *n* = 13 (5th ST), *n* = 6 (6th ST)) (right panel). Symbols represent results from individual mice, horizontal lines indicate mean ± SEM. ^***^p < 0.001, unpaired *t*-test with Welch’s correction. **b** Surface marker expression on transformed hSTAT5B^N642H^ NKT cells was analyzed by flow cytometry. Representative histograms from BM NKT cells of one diseased NSG mouse (4th ST) are shown (unfilled histogram: negative staining control; filled histogram: surface staining). Numbers depict the mean of MFI (median fluorescence intensity, normalized to negative staining control) ± SEM from three diseased mice (4th ST). **a**, **b** Analysis was performed when the diseased mice reached the humane endpoint. **c** NSG mice were transplanted with BM containing 1 × 10^6^ hSTAT5B^N642H^ NKT cells (from 5th ST) and treated with Ruxolitinib (RUXO) (85 mg/kg body weight, twice daily) or vehicle control (Nutella^®^) (*n* = 4 per treatment), starting 1 day after NKT cell transplant for 21 days (one experiment). Representative images of liver and spleen to body weights from untreated control and RUXO-treated mice are depicted; line denotes 1 cm (left panel). Relative liver and spleen weights were analyzed in the mice after 3 weeks of treatment (right panel). Symbols represent results from individual mice, horizontal lines indicate mean ± SEM. ^**^p < 0.01, ^***^p < 0.001, unpaired *t*-test

To investigate, whether hSTAT5B^N642H^ NKT cells are indeed transformed and fulfill the criteria of being leukemic, we started to perform serial whole BM transplants from the earlier diseased recipient #1 (survival: 133 d) with the presumably more aggressive disease. To do so, we transplanted BM containing 1 × 10^6^ NKT cells into immune-deficient Rag2^−/−^γc^−/−^ or NSG-recipient mice. This serial transplantation approach was continued for a total of six rounds. The first two rounds were performed to maintain and amplify the NKT cell disease. We confirmed the presence of the hSTAT5B^N642H^ transgene in a diseased mouse (Fig. S1J). Upon the 3rd to the 6th round of serial transplant, we characterized the manifested disease as an aggressive leukemia accompanied by hepatosplenomegaly (Fig. 2a), increased white blood cell (WBC) counts (Fig. S2A) and a high frequency of NKT cells in blood, BM, spleen, and liver (Fig. S2C, D) with a mean survival of 17.6 days (Fig. S2B). Infiltration of leukemic cells into various organs was confirmed by histological analysis (Fig. S2E). In the 6th round, a titration of transplanted NKT cell numbers was performed. Disease severity at the end point was comparable, while a delay of 3 days occurred between each titration step (Fig. 2a, Fig. S2A–D). Despite the increased disease latency in recipients #2 (survival: 294 d) and #3 (survival: 301 d), the disease could also be serially transplanted giving rise to a similarly aggressive disease (Fig. S3A–H) as for recipient #1.

A more detailed characterization of surface markers on transformed NKT cells from the serially transplanted disease of recipient #1 verified expression of the T cell receptor (TCR) β chain, while CD4, CD8, and TCRγδ expression was not detected. Leukemic cells also stained positive for CD122 and the NK cell markers NKp46 and DX5. No interaction with the CD1d tetramer was observed (Fig. 2b). This surface marker profile corresponds to previously described CD1d-independent NKT cells [8, 9], which were recently identified as a source for CD3^+^NK1.1^+^ T-cell large granular lymphocyte (T-LGL) leukemia in IL-15 transgenic mice [10]. In analogy to our NKT cell leukemia model, human CD56^+^ T-LGL leukemia cells were also shown to express NKp46 [10]. Additionally, CD3^+^CD56^+^ blasts in human T-LGL leukemia express CD8 [4]. Interestingly, we found CD8 expressed on a subset of hSTAT5B^N642H^ NKT leukemia cells in the two later diseased recipients #2 and #3, but not in recipient #1 (Table S1).

Furthermore, the serially transplanted leukemic NKT cells from recipient #1 were largely negative for activating and inhibitory NK cell receptors, except for Ly49G2 and KLRG1 (Fig. S4A), but stained positive for CD43, CD44, and CD69, while lacking CD62L (Fig. S4A). In summary, this surface receptor expression profile is consistent with an activated phenotype comparable to the leukemic blasts found in IL-15 transgenic mice [11]. The fact that IL-15 transgenic mice develop NK1.1^+^ T-LGL (NKT) or NK cell leukemia [10, 11] supports the importance of the IL-15-STAT5 axis for the transformation of subsets of innate lymphocytes. The occurrence of leukemia with an NKT cell profile in our model suggests that additional IL-15-derived signals, independent of STAT5, may be required for NK cell transformation. In contrast to IL-15 transgenic mice, in which transformation is restricted to innate-like lymphocytes, NKp46^+^ NKT cell disease required the absence of classical CD8^+^ T cells bearing mutant hSTAT5B in vivo. As NKp46^+^ NKT cells represent a minor population [10], the higher number of CD8^+^ T cells may simply outcompete potentially transformed NKT cells. Alternatively, it is possible that CD8^+^ T cells actively suppress the development of NKT cell tumors providing a hostile tumor microenvironment.

hSTAT5B^N642H^-driven CD8^+^ T cell disease is susceptible to JAK1/JAK2 inhibition by Ruxolitinib treatment [7]. As JAK inhibitors have also been suggested as therapeutic strategy for NK/T-cell lymphoma and aggressive NK cell leukemia [6, 12], we transplanted NSG mice with hSTAT5B^N642H^ NKT leukemia cells and treated them with ruxolitinib for 3 weeks. Treatment attenuated disease severity and decreased hepatosplenomegaly and WBC counts compared to the control group (Fig. 2c and Fig. S4B).

Although initial attempts to grow transformed NKT cells in vitro failed, after 6–8 weeks we detected outgrowth of hSTAT5B^N642H^ NKT cell lines from cultured hepatic leukocytes of three out of nine NSG mice from different serial transplant rounds from recipient #1 (Table S2, Fig. S5A). Two cell lines, derived from the same mouse cultured without or with IL-2 (4165_1 and 4165_2, respectively), were treated with Ruxolitinib and remained sensitivity towards it (IC_50_ of 83 and 72.5 nM, respectively) (Fig. S5B). Furthermore, these cell lines could give rise to leukemia when injected into immune-competent Ly5.1/CD45.1^+^ mice (Fig. S5C–E).

In this study, we identify STAT5B^N642H^ as an oncogenic driver in innate-like lymphocytes. Our novel NKT leukemia model, which is serially transplantable and inducible by frozen material or from cell lines, recapitulates human CD56^+^ LGL leukemia and provides new means for translational research. It will allow a deeper understanding of mechanisms driving NKT leukemia progression and maintenance as well as facilitate finding new therapeutic options. This is of vital importance as the STAT5B^N642H^ mutation in CD56^+^ LGL leukemia patients is associated with a particularly aggressive chemo-refractory phenotype [4].

## Supplementary information

Supplemental Figures and Tables

Supplementary Information - Material and Methods
