# Supplementary material for: STAT5BN642H drives transformation of NKT cells: a novel mouse model for CD56+ T-LGL leukemia
Source: Leukemia. 2019 Apr 9;33(9):2336–40. doi: 10.1038/s41375-019-0471-3 (PMC6756040; doi:10.1038/s41375-019-0471-3)
Supplement: Supplementary file 1 — Supplemental Figures and Tables [file 41375_2019_471_MOESM1_ESM.pdf]

Supplemental Figures and Tables

Supplemental Figure 1. hSTAT5B^{N642H} induces temporary NK cell expansion upon BM transplant in contrast to hSTAT5B, while hSTAT5B and hSTAT5B^{N642H} similarly promote NK cell maturation.

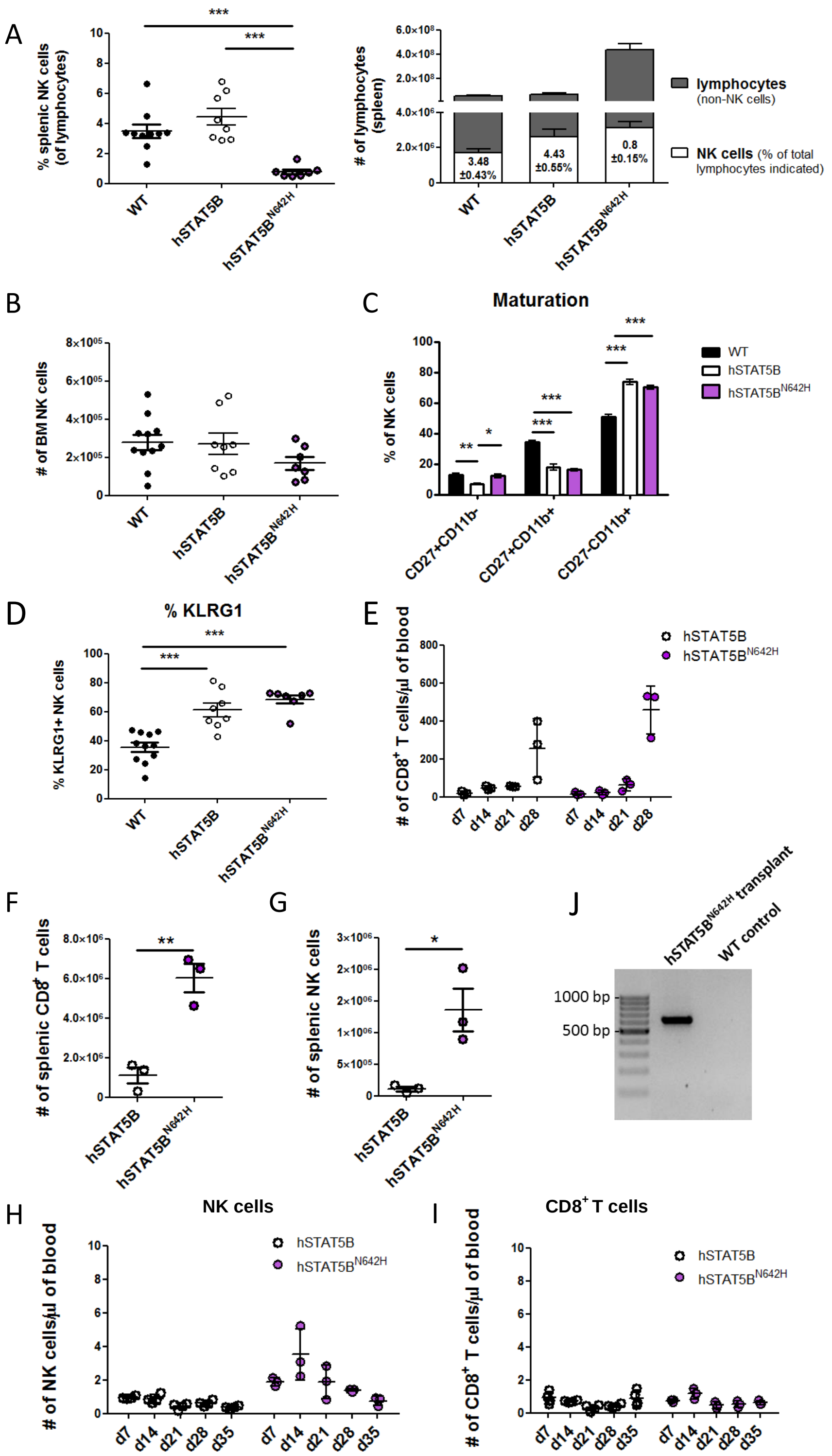

(A) Frequency of splenic NK cells ($CD3^-NK1.1^+NKp46^+$) (left panel) and absolute numbers of splenic lymphocytes (right panel), indicating the proportion of NK cells (with mean ± SEM NK cell percentage of total lymphocytes according to left panel) and non-NK cells, were determined in WT, hSTAT5B and $hSTAT5B^{N642H}$ mice by flow cytometry (n=10 (WT), n=8 (hSTAT5B), n=7 ($hSTAT5B^{N642H}$) pooled from 4 independent experiments). Symbols represent results from individual mice, horizontal lines or bar graphs indicate mean ± SEM. $^{***}p< 0.001$, one-way ANOVA. (B) Absolute numbers of BM NK cells ($Lin^-CD122^+NK1.1^+NKp46^+$) were determined in WT, hSTAT5B and $hSTAT5B^{N642H}$ mice by flow cytometry (n=11 (WT), n=8 (hSTAT5B), n=7 ($hSTAT5B^{N642H}$) pooled from 4 independent experiments). Symbols represent results from individual mice, horizontal lines indicate mean ± SEM. (C-D) Frequency of $CD27^+CD11b^-$, $CD27^+CD11b^+$ and $CD27^-CD11b^+$ maturation stages of NK cells ($CD3^-NK1.1^+NKp46^+$) (C) or frequency of $KLRG1^+$ NK cells (D) were analyzed in the spleen of WT, hSTAT5B and $hSTAT5B^{N642H}$ mice by flow cytometry (n=10 (WT), n=8 (hSTAT5B), n=7 ($hSTAT5B^{N642H}$) pooled from 4 independent experiments). Symbols represent results from individual mice, horizontal lines or bar graphs indicate mean ± SEM. $^{*}p< 0.05$, $^{**}p< 0.01$, $^{***}p< 0.001$, one-way ANOVA. (E-G) CD3-depleted BM from hSTAT5B or $hSTAT5B^{N642H}$ mice was transplanted into $Rag2^{-/-}\gamma c^{-/-}$ recipient mice (n=3) (Fig. 1B). $CD8^+$ T cell numbers were monitored in the blood weekly for 4 weeks (E). $CD8^+$ T cell (F) and NK cell (G) numbers in the spleen of recipients were analyzed 31 days after transplant by flow cytometry. Symbols represent results from individual mice, horizontal lines indicate mean ± SEM. One representative experiment from two independent experiments is shown. (F,G) $^{*}p< 0.05$, $^{**}p< 0.01$, unpaired t-test. (H, I) Lineage $(Lin)^-Sca1^-c\text{-}Kit^-CD127^-CD8^-$ cells were sorted from BM of hSTAT5B or $hSTAT5B^{N642H}$ mice and $1*10^5$ cells were injected into $Rag2^{-/-}\gamma c^{-/-}$ recipient mice (n=3) (Fig. 1C). Numbers of NK (H) and $CD8^+$ T cells (I) were monitored in the blood of the transplanted mice weekly for 5 weeks. Symbols represent results from individual mice, horizontal lines indicate mean ± SEM. (J) $hSTAT5B^{N642H}$ transgene expression was confirmed in the blood of an $hSTAT5B^{N642H}$-transplanted mouse from the 2nd round of serial transplant from recipient #1 by genotyping PCR. WT control = genomic DNA from a WT mouse.

Supplemental Figure 2. hSTAT5B^{N642H} induces NKT cell leukemia, which is serially transplantable.

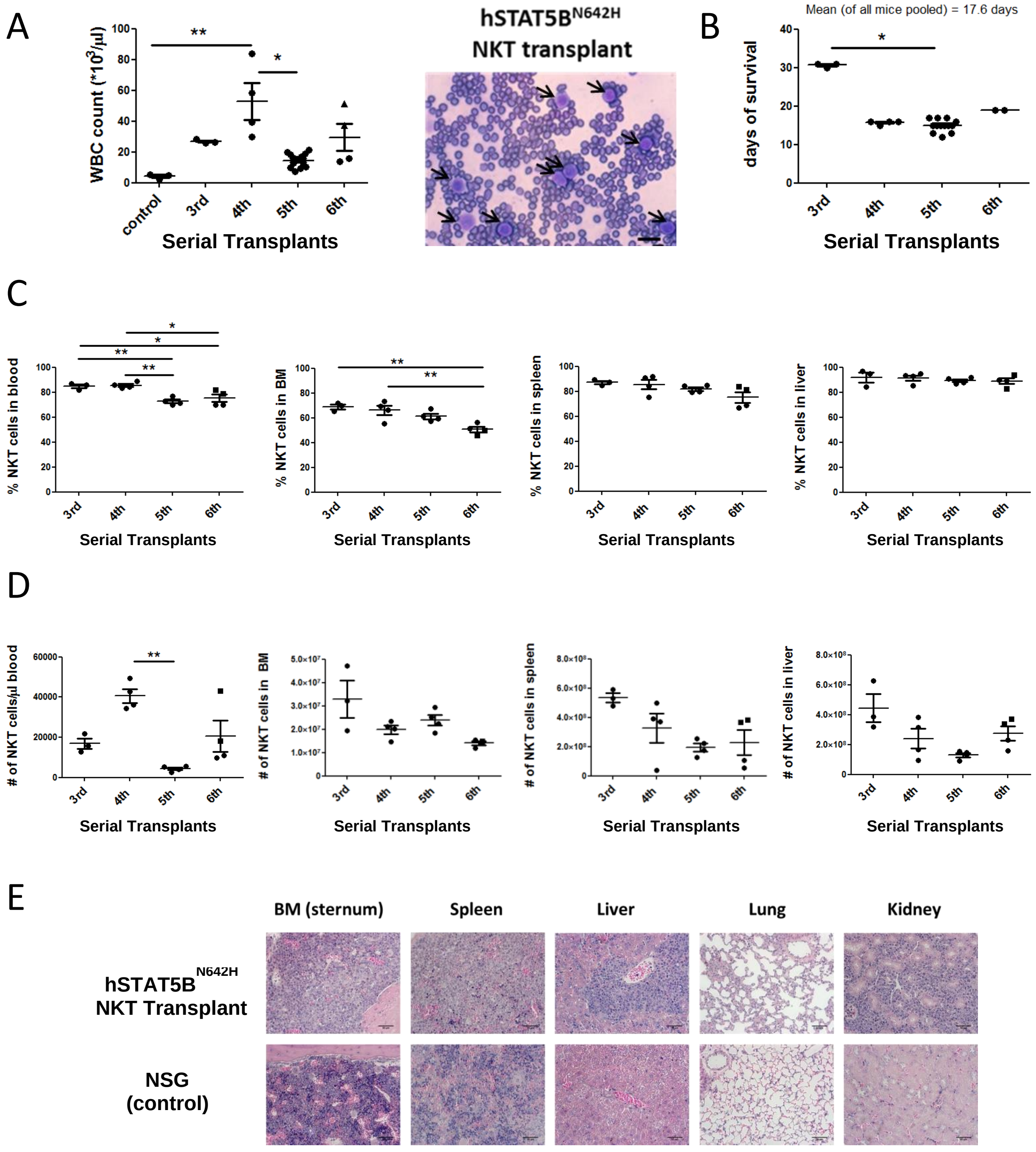

(A-E) BM from diseased hSTAT5B^{N642H}-transplanted recipient #1 was serially transplanted into Rag2$^{-/-}$γc$^{-/-}$ or NSG recipient mice for six rounds. Whole BM, containing 1*10^6 transformed NKT cells, was transplanted. Of note, in the 6th round a titration of the number of transplanted NKT cells was performed, with two mice each receiving BM containing 1*10^6 (circle), 0.3*10^6 (rectangle) or 0.1*10^6 (triangle) NKT cells. (A) WBC counts were measured by animal blood counter VetABC in serially transplanted diseased mice from 3rd to 6th round,

compared to non-transplanted NSG mice (left panel). Representative blood smear of an hSTAT5B^{N642H} NKT cell-transplanted mouse from the 6th round is shown; lines represent 20µm. Arrows indicate blasts (right panel). (B) Days of survival of transplanted recipient mice are shown for the 3rd to 6th round. For the 6th round only results from mice transplanted with BM containing 1*10^{6} NKT cells are shown. (C, D) Percentage (C) and absolute numbers (D) of transformed NKT cells were analyzed in blood, BM, spleen and liver of diseased mice upon serial transplantation by flow cytometry. (A-D) Symbols represent results from individual mice, horizontal lines indicate mean ± SEM. *p< 0.05, **p< 0.01, Kruskal-Wallis test (A,B,D), one-way ANOVA (C). (E) Histological analysis of BM (sternum), spleen, liver, lung and kidney was performed using H&E staining. Representative images of an hSTAT5B^{N642H} NKT cell-transplanted mouse from the 6th round and a non-transplanted NSG control are shown. Line denotes 50µm. (A-E) Analysis was performed when the diseased mice reached the humane endpoint.

Supplemental Figure 3. hSTAT5B^{N642H}-driven NKT cell disease is serially transplantable

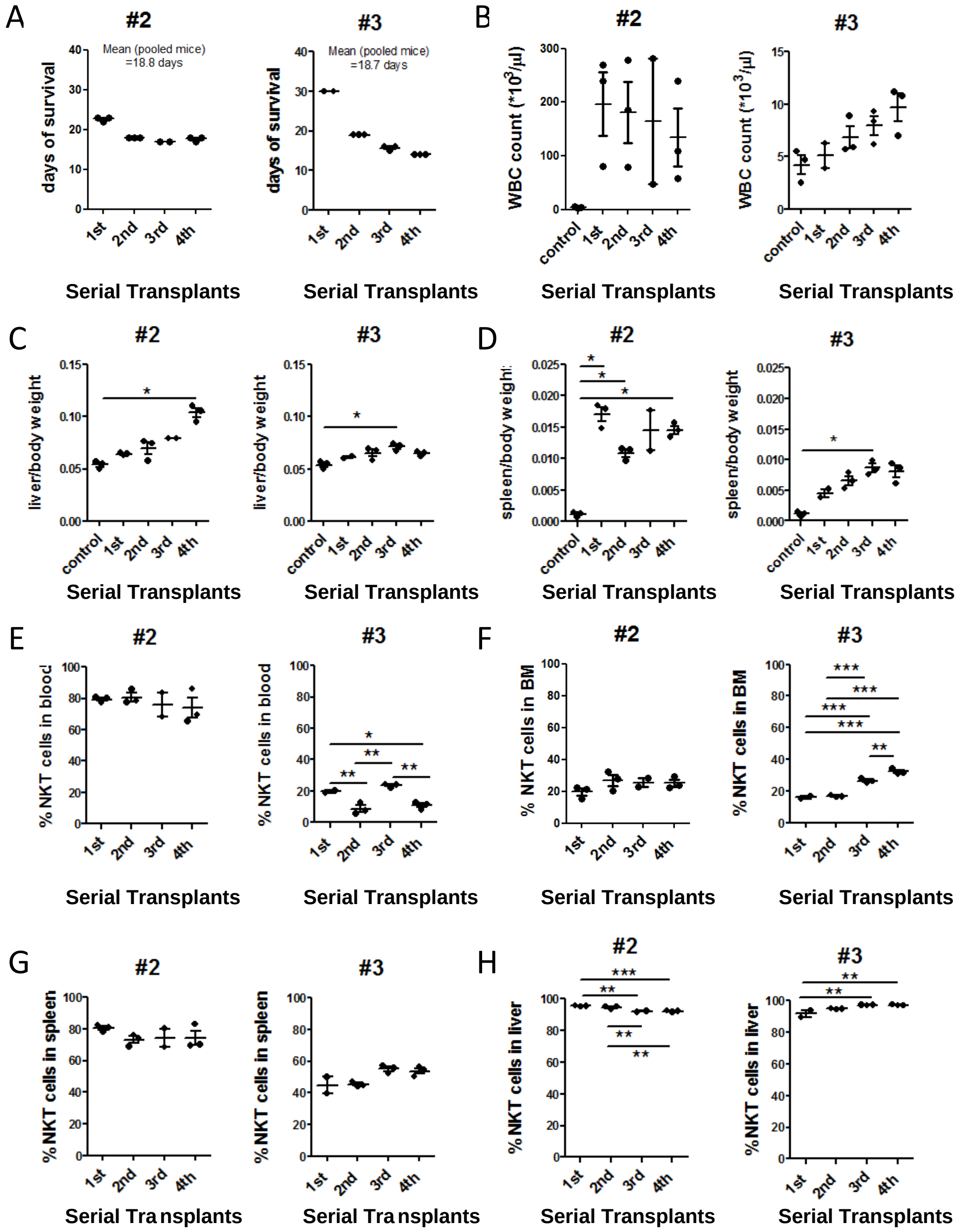

(A-H) BM from later diseased hSTAT5B^{N642H}-transplanted recipient mice (#2 and #3) was serially transplanted into NSG mice for four rounds (n=2-3/round). Whole BM, containing $0.3*10^6$ transformed NKT cells, was transplanted. Days of survival (A), WBC counts (B), relative liver (C) and spleen (D) to body weights and frequency of transformed NKT cells in blood (E), BM (F), spleen (G) and liver (H) measured by flow cytometry are shown for diseased recipients #2 (left panels) and #3 (right panels) upon serial transplantation. Control = non-transplanted NSG mice. Symbols represent results from individual mice, horizontal lines

indicate mean ± SEM. $^{***}p < 0.001$, $^{**}p < 0.01$, $^{*}p < 0.05$, Kurskal-Wallis test (A-B), unpaired t-test with Welch's correction (C-D), one-way ANOVA (E-H). (A-H) Analysis was performed when the diseased mice reached the humane endpoint.

Supplemental Figure 4. hSTAT5B^{N642H} induces NKT cell leukemia with an activated phenotype, which is sensitive to Ruxolitinib treatment.

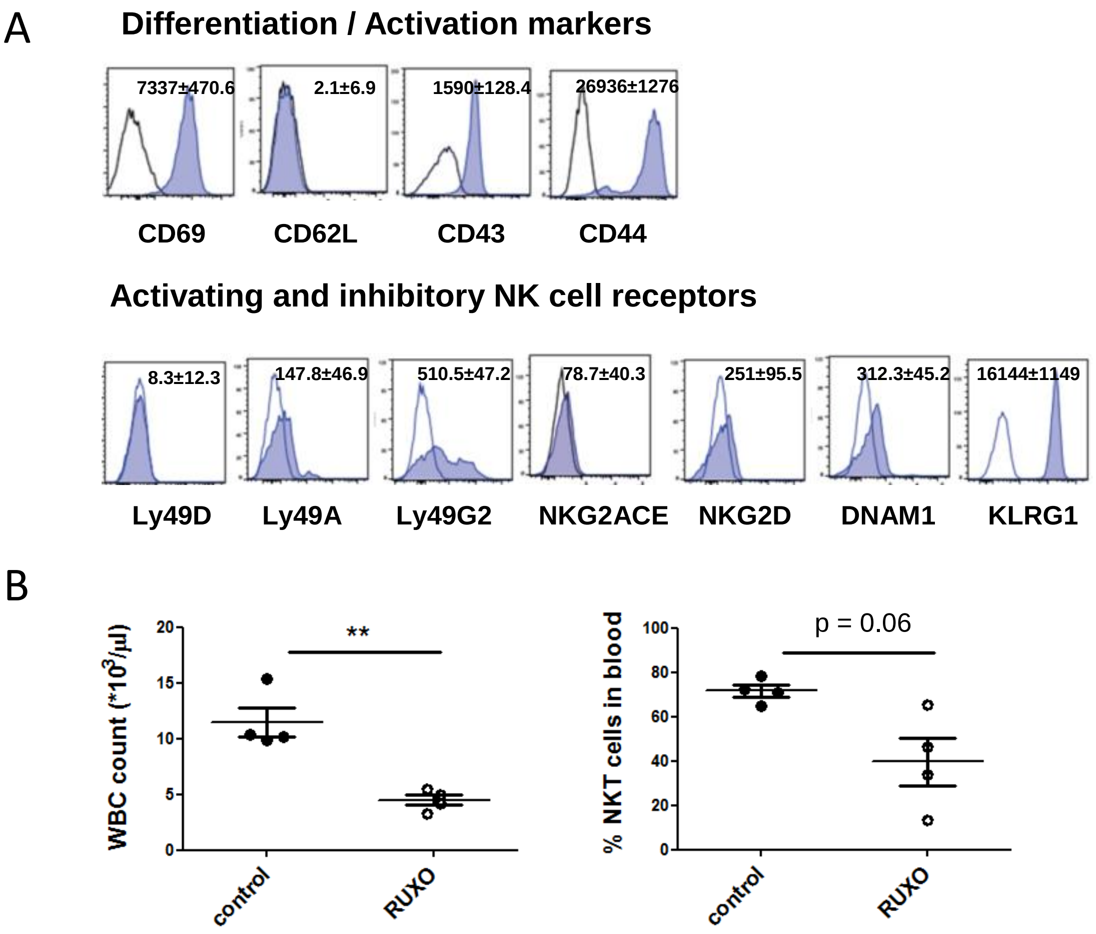

(A) Surface marker expression on transformed hSTAT5B^{N642H} NKT cells was analyzed by flow cytometry. Representative histograms from one diseased NSG mouse (4th round of serial transplant from recipient #1) are shown (unfilled histogram: negative staining control; filled histogram: surface staining). Numbers depict the mean of MFI (median fluorescence intensity, normalized to negative staining control) ± SEM from three mice (4th round of serial transplant). (B) NSG mice were transplanted with BM containing $1*10^6$ hSTAT5B^{N642H} NKT cells (from 5th serial transplant from recipient #1) and treated with Ruxolitinib (RUXO) (85mg/kg bodyweight, twice daily) or vehicle control (Nutella®) (n=4 per treatment), starting one day after transplant for 21 days (one experiment). WBC counts (left panel) and

percentage of NKT cells in the blood (right panel) upon 21 days of treatment are shown. Symbols represent results from individual mice, horizontal lines indicate mean ± SEM. $^{**}p < 0.01$, unpaired t-test (with Welch's correction for right panel).

Supplemental Figure 5. Establishment of hSTAT5B^{N642H} NKT cell leukemia lines.

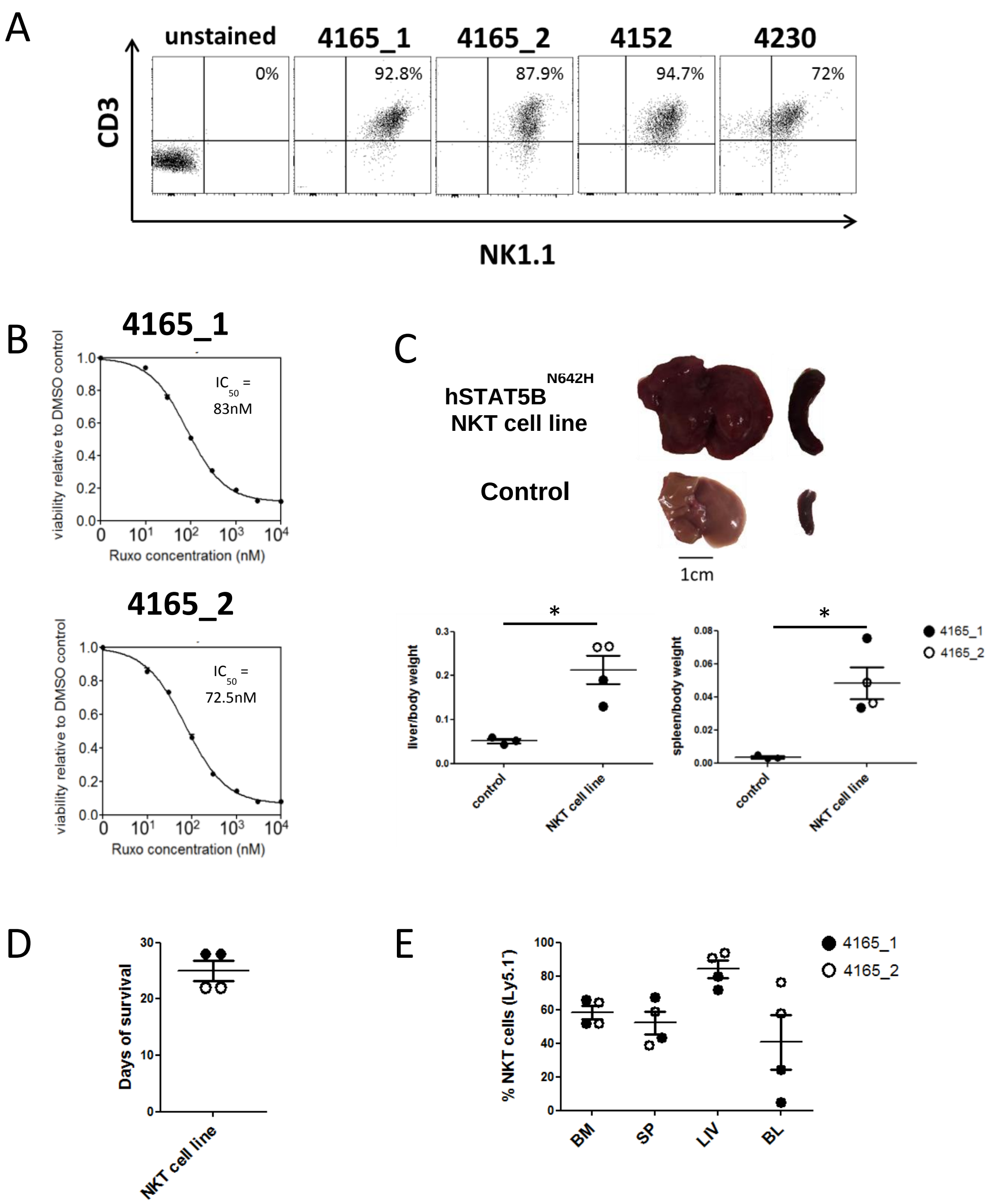

(A) NKT cell lines were analyzed for CD3 and NK1.1 expression by flow cytometry. Dot plots are shown. Unstained cells are shown as a control. (B) 4165_1 and 4165_2 NKT cell lines (Tab. S2) were treated with Ruxolitinib (Ruxo) at indicated concentrations or DMSO control for three days and viability was analyzed by CellTiter-Glo assay. Dose-response

curves and IC_{50} values are shown (one experiment; n= 3 technical replicates). (C-E) $0.5*10^6$ 4165_1 and 4165_2 cells were intravenously injected into immune-competent Ly5.1/CD45.1$^+$ recipient mice (n=2 per cell line). (C) Representative images of liver and spleen from one diseased Ly5.1 recipient compared to a non-injected control mouse (upper panel) are shown. Line denotes 1cm. Relative liver and spleen to body weights of diseased mice (lower panel) are shown (filled circles = mice injected with 4165_1; unfilled circles = mice injected with 4165_2). Days of survival (D) and frequency of hSTAT5B^{N642H} NKT cells (Ly5.1$^-$ CD3$^+$NK1.1$^+$) in BM, spleen (SP), liver (LIV) and blood (BL) measured by flow cytometry (E) are shown. (C-E) Symbols represent results from individual mice, horizontal lines indicate mean ± SEM. (C) *$p < 0.05$, unpaired t-test with Welch's correction. (C-E) Analysis was performed when the diseased mice reached the humane endpoint.

Supplemental Table 1.

Days of survival after transplant, relative spleen and liver to body weights, WBC counts, surface marker expression and frequency of NKT cells in indicated organs are depicted for the diseased hSTAT5B^{N642H}-transplanted Rag2$^{-/-}\gamma c^{-/-}$ recipient mice (#1-3) (as described in Fig. 1C) and one non-diseased hSTAT5B-transplanted control mouse.

**Mouse**		**#1** (hSTAT5B^{N642H} transplant)	**#2** (hSTAT5B^{N642H} transplant)	**#3** (hSTAT5B^{N642H} transplant)	**control** (**hSTAT5B transplant)**
**Survival (d)**		133	294	301	no signs of sickness (analysed after 309 days)
**Relative spleen weight (/body weight)**		0.003	0.011	0.001	0.002
**Relative liver weight (/body weight)**		*no data*	0.094	0.053	0.05
**WBC** (*10^3/µl)		1.6	5.2	4.4	4.8
**Surface markers**	**CD3**	pos.	pos.	pos.	-
	**NK1.1**	pos.	pos.	pos.	-
	**NKp46**	pos.	pos.	pos.	-
	**CD4**	neg.	neg.	neg.	-
	**CD8**	neg.	neg/pos. (20% in BM, 60-80% in spleen and liver)	neg/pos. (<5% in BM, 30-40% in spleen and liver)	-
**% of NKT cells in organs**	**blood**	24.3	4.51	4	0
	**BM**	4.22	15	7.38	0
	**spleen**	52.9	37.2	*no data*	0
	**liver**	90.6	91.9	27.4	0

Supplemental Table 2.

Whole splenocytes (SP) or hepatic leukocytes (LIV) (containing 70-95% NKT cells) were isolated from the indicated NKT cell-diseased recipient mice (2-3 NSG mice from 3rd to 6th serial transplant from recipient #1) and cultured with or without 200U/ml IL-2. NKT cell lines could be established (outgrowth = Y) after 6-8 weeks from three out of nine mice and were derived from hepatic leukocytes upon culture with IL-2 with the exception of 4165, where outgrowth was also detected without IL-2 (4165_1 line). Y = yes (outgrowth), N = no.

Mouse #	Serial transplant	organ	culture	outgrowth
**4165**	3rd	SP	no IL2	N
			200U/ml IL2	N
		LIV	no IL2	Y (4165_1)
			200U/ml IL2	Y (4165_2)
**4166**		SP	no IL2	N
			200U/ml IL2	N
		LIV	no IL2	N
			200U/ml IL2	N
**4151**	4th	SP	no IL2	N
			200U/ml IL2	N
		LIV	no IL2	N
			200U/ml IL2	N
**4152**		SP	no IL2	N
			200U/ml IL2	N
		LIV	no IL2	N
			200U/ml IL2	Y (4152)
**4153**		SP	no IL2	N
			200U/ml IL2	N
		LIV	no IL2	N
			200U/ml IL2	N
**4194**	5th	SP	200U/ml IL2	N
		LIV	200U/ml IL2	N
**4197**		SP	200U/ml IL2	N
		LIV	200U/ml IL2	N
**4229**	6th	SP	no IL2	N
			200U/ml IL2	N
		LIV	no IL2	N
			200U/ml IL2	N
**4230**		SP	no IL2	N
			200U/ml IL2	N
		LIV	no IL2	N
			200U/ml IL2	Y (4230)
