# Supplementary material for: STAT5BN642H drives transformation of NKT cells: a novel mouse model for CD56+ T-LGL leukemia
Source: Leukemia. 2019 Apr 9;33(9):2336–40. doi: 10.1038/s41375-019-0471-3 (PMC6756040; doi:10.1038/s41375-019-0471-3)
Supplement: Supplementary file 2 — Supplementary Information - Material and Methods [file 41375_2019_471_MOESM2_ESM.pdf]

Supplementary Information

Material and Methods

Mice

hSTAT5B (B6N-Tg(STAT5B)731Biat) and hSTAT5B^{N642H} (B6N-Tg(STAT5BN/H)726Biat) mice were described previously[1]. These transgenic mice were bred heterozygous for the transgene on C57BL/6N background. Wild-type (WT) control mice were littermates from these lines. NSG (NOD.Cg-Prkdc^{scid} Il2rg^{tm1}Wjl/SzJ)[2], Rag2$^{-/-}$γc$^{-/-}$ (C.Cg-Rag2^{tm1Fwa}-Il2rg^{tm1Wji})[3] and Ly5.1 (B6.SJL-Ptprc^{a})[4] recipient mice were in-house bred and maintained under specific pathogen-free conditions at the University of Veterinary Medicine, Vienna according to Federation for Laboratory Animal Science Associations (FELASA) guidelines (2014). The animal experiments were approved by the Ethics and Animal Welfare Committee of the University of Veterinary Medicine Vienna and the national authority (Austrian Federal Ministry of Science and Research) according to §§ 26ff. of Animal Experiments Act, Tierversuchsgesetz 2012—TVG 2012, under licenses BMWFW-68.205/0103-WF/V/3b/2015 and BMWFW-68.205/0112-WF/V/3b/2016 and were conducted according to the guidelines of FELASA and ARRIVE. Sex and age-matched (7-9 weeks) WT, hSTAT5B and hSTAT5B^{N642H} mice were used for data presented in Fig. 1 and Fig. S1. Recipient mice for serial transplantation were age-matched (8-12 weeks) and both sexes were used.

Hematocytometry, organ preparation and flow cytometry

Mouse blood was collected in EDTA-tubes from the facial vein or from euthanized mice via cardiac puncture. White blood cell (WBC) count was measured using an animal blood counter (sciI Vet abc). For blood analysis by flow cytometry, erythrocytes were lysed using BD FACS Lysing Solution according to manufacturer's protocol (BD Bioscience, San Diego, CA, USA). Single cell suspensions were prepared from spleen, liver and bone marrow (BM). Isolation of hepatic leucocytes was performed by separation with 37.5% percoll (GE Healthcare, Chicago, Illinois, USA). Prior to flow cytometric analysis, lysis of erythrocytes from single cell suspension was performed with Red blood cell Lysis Buffer (10 mM KHCO3 and 75 mM NH4Cl, pH 7.4).

The antibodies (clones) targeting following proteins were purchased from eBioscience (San Diego, CA, USA): B220 (RA3-6B2), CD3 (17A2), CD3e (145-2C11), CD11b (M1/70),

CD16/CD32 (93), CD19 (eBio1D3), CD49b (DX5), CD122 (5H4), CD127 (A7R34), CD226 (10E5), CD27 (LG.7F9), c-Kit (2B8), Gr-1 (RB6-8C5), KLRG1 (2F1), Ly49A (A1), Ly49G2 (eBio4D11), NKG2A/C/E (20d5), NKG2D (CX5), NKp46 (29A1.4), NK1.1 (PK136), Sca-1 (D7), Ter119 (TER-119); from Biolegend (San Diego, CA, USA): CD45.1 (A20), TCRβ (H57-597), TCRγδ (UC7-13D5), Streptavidin-APC/Cy7; or from BD Pharmingen (San Diego, CA, USA): CD4 (GK1.5), CD8 (53-6.7), CD43 (S7), CD44 (IM7), CD62L (MEL-14), CD69 (H1.2F3), Ly49D (4E5). Lineage panel included CD3, B220, Gr-1, CD11b and Ter119. The PBS-57 loaded CD1d tetramer was provided by the NIH Tetramer Core Facility. Flow cytometry experiments were performed on a BD FACSCanto II (BD Bioscience) and analyzed using BD FACSDiva V8.0 or FlowJo V10 software. Cells were sorted using a BD FACSAria machine.

Absolute cell numbers were determined by flow cytometry by adding CountBright^{TM} Absolute Counting Beads (Thermo Fisher Scientific, Waltham, Massachusetts, USA).

BM transplant and serial transplant experiments

$CD3^+$ T cell depletion of BM from hSTAT5B or hSTAT5B^{N642H} mice was performed with MagniSort Mouse CD3 Positive Selection Kit according to manufacturer's protocol (eBioscience). CD3-depleted BM from one hSTAT5B or hSTAT5B^{N642H} mouse was transplanted into three Rag2$^{-/-}$γc$^{-/-}$ recipient mice (for transplants described in Fig. 1B and S1E-G). For transplants described in Fig. 1C and S1H-I, hSTAT5B BM was lineage-depleted (using MagniSort Mouse Hematopoietic Lineage Depletion Kit (eBioscience)) and hSTAT5B^{N642H} BM was CD3-depleted prior to sort of Lineage (Lin)$^-$ (CD3/B220/Ter119/Gr1/CD11b)CD127$^-$CD8$^-$Sca1$^-$ckit$^-$ cells. $1*10^5$ sorted cells were transplanted into each of three Rag2$^{-/-}$γc$^{-/-}$ recipient mice.

For serial BM transplantation of hSTAT5B^{N642H} NKT cell-driven disease established in recipient mouse #1 (Fig. 2 and S2), BM was isolated from diseased mice and frequency of transformed NKT cells was determined by flow cytometry. Whole BM, containing $1*10^6$ NKT cells was serially transplanted into Rag2$^{-/-}$γc$^{-/-}$ or NSG recipient mice for a total of six rounds. From the 3rd round on only NSG mice were used for serial transplants. Of note, in the 6th round a titration of the number of transplanted NKT cells was performed, with two NSG mice receiving $1*10^6$ NKT cells, two NSG mice receiving $0.3*10^6$ NKT cells and two NSG mice receiving $0.1*10^6$ NKT cells. However, disease severity at the end point was similar

independent of initially injected cell numbers. Therefore, data from all mice of the 6th round were pooled for analysis of disease severity, except for depicting days of survival (Fig. S2B), where only data from mice injected with 1*10^6 NKT cells were included. The mice were sacrificed at the first signs of disease (humane endpoint). For serial transplantation of NKT cell disease established in recipient mice #2 and #3 (Fig. S3), BM containing 0.3*10^6 NKT cells was serially transplanted into NSG recipient mice for a total of four rounds.

NKT cell lines

Whole splenocytes or hepatic leukocytes (containing 70-95% NKT cells) were isolated from NKT cell-diseased mice (2-3 NSG mice from 3rd to 6th serial transplant from recipient #1) and cultured with or without 200 U/ml human recombinant (rh)IL-2 (Proleukin, Novartis, Basel, Switzerland) at a density of 2*10^6 cells/ml in RPMI1640 (Sigma, St. Louis, Missouri, USA) medium containing 20% FCS (Bio & Sell, Feucht, Germany), 100 U/mL penicillin, 100 mg/mL streptomycin (Sigma), and 50µM 2-mercaptoethanol (Sigma). Cells were monitored for outgrowth. Established NKT cell lines could continuously be maintained *in vitro* for an observation period of at least 4-6 months.

***In vivo* Ruxolitinib treatment**

NSG mice were transplanted with BM containing 1*10^6 hSTAT5B^{N642H} NKT cells (from 5th round of serial transplant from recipient #1) and treated with Ruxolitinib (Jakavi®, Novartis) (85mg/kg body weight) as a powder in a Nutella® ball or Nutella® alone (control) twice daily, starting one day after transplant for 21 days.

***In vitro* Ruxolitinib treatment (Viability assay)**

hSTAT5B^{N642H} NKT cell lines (2 × 10^4 cells) were seeded in triplicate in 96-well plates. Ruxolitinib (Selleckchem, Houston, Texas, USA) was dissolved in DMSO (Sigma), added at different concentrations (as indicated in Fig. S5B) and incubated for 72 hours. DMSO (100% viability) and 10µM bortezomib (Selleckchem) (0% viability) were used as a negative and positive control, respectively. CellTiter-Glo reagent (Promega, Madison, Wisconsin, USA) was used to determine viability and luminescence was measured on an EnSpire plate reader (PerkinElmer, Waltham, Massachusetts, USA). IC_{50} values were determined by nonlinear regression using GraphPad Prism Software (GraphPad Software Inc., San Diego, CA, USA).

PCR

Genomic DNA was isolated from blood of an hSTAT5B^{N642H}-transplanted recipient mouse (from 2nd round of serial transplant from recipient #1) and transgene expression was analyzed by genotyping PCR (forward primer: ACGCAGGACACAGAGAATGAG; reverse primer: GTGATGGTGGCGTTGACCTC)[1]. Genomic DNA from a wild-type mouse served as negative control.

Histology

Organs were fixed in 4% phosphate buffered formaldehyde solution (Roti®Histofix, Carl Roth, Karlsruhe, Germany), dehydrated, embedded and cut (4 µm). Paraffin-embedded sections were stained with hematoxylin and eosin (H&E) according to standard histological procedures. Blood smears were stained using a Hemacolor® Rapid staining of blood smears kit (Sigma, St. Louis, Missouri, USA). Images were taken using an Olympus IX71 microscope and CellSens Dimension Software (Olympus, Tokyo, Japan).

Statistical analysis

Two-sided unpaired t-test was performed with or without Welch's correction depending on homogeneity of variance and Bonferroni correction was performed for multiple testing. One-way ANOVA was followed by Tukey's multiple comparison test. Kruskal–Wallis test was followed by Dunn's test. All tests were performed using GraphPad Prism version 5.00 (GraphPad Software Inc., San Diego, CA, USA). Count data were log-transformed for statistical analysis. The level of significance is indicated for each experiment (* $p < 0.05$; ** $p < 0.01$; *** $p < 0.001$).

References

1. Pham HTT, Maurer B, Prchal-Murphy M, Grausenburger R, Grundschober E, Javaheri T, et al. STAT5B N642H is a driver mutation for T cell neoplasia. *J Clin Invest*. 2018;128(1):387–401.

2. Ito M, Hiramatsu H, Kobayashi K, Suzue K, Kawahata M, Hioki K, et al. NOD/SCID/γcnull mouse : an excellent recipient mouse model for engraftment of human cells. *Blood*. 2002;100(9):3175–82.

3. Cao X, Shores EW, Hu-li J, Anver MF, Kelsall BL, Sarah MR, et al. Defective Lymphoid Development in Mice Lacking Expression of the Common Cytokine Receptor y Chain. *Immunity*. 1995;2:223–38.

4. Zebedee SL, Barritt DS,Raschke WC. Comparison of Mouse Ly5 a and Ly5 b Leucocyte Common Antigen Alleles. Dev Immunol. 1991;1(4):243-54.
